# Whole-Genome Shotgun Sequence of the Keratinolytic Bacterium Lysobacter sp. A03, Isolated from the Antarctic Environment

**DOI:** 10.1128/genomeA.00246-15

**Published:** 2015-04-02

**Authors:** Jamile Queiroz Pereira, Adriana Ambrosini, Fernando Hayashi Sant’Anna, Michele Tadra-Sfeir, Helisson Faoro, Fábio Oliveira Pedrosa, Emanuel Maltempi Souza, Adriano Brandelli, Luciane M. P. Passaglia

**Affiliations:** a Departamento de Genética, Instituto de Biociências, Universidade Federal do Rio Grande do Sul (UFRGS), Porto Alegre, RS, Brazil; b Instituto de Ciências e Tecnologia de Alimentos, Universidade Federal do Rio Grande do Sul (ICTA-UFRGS), Porto Alegre, RS, Brazil; c Departamento de Bioquímica e Biologia Molecular, Universidade Federal do Paraná (UFPR), Curitiba, PR, Brazil

## Abstract

Lysobacter sp. strain A03 is a protease-producing bacterium isolated from decomposing-penguin feathers collected in the Antarctic environment. This strain has the ability to degrade keratin at low temperatures. The A03 genome sequence provides the possibility of finding new genes with biotechnological potential to better understand its cold-adaptation mechanism and survival in cold environments.

## GENOME ANNOUNCEMENT

Lysobacter is a genus of Gram-negative bacteria first described in 1978 (1) that belongs to the family Xanthomonadaceae, within the Gammaproteobacteria. They are characterized by gliding motility, a high G+C content, and the production of a broad range of proteases and antibiotics, thus representing a source of biocontrol agents (2).

The Antarctic strain A03 was isolated from decomposing-penguin feathers collected on King George Island, Antarctica, and was identified as a Lysobacter sp. by both 16S rRNA and 16S-23S rRNA intergenic transcribed spacer gene sequencing. The isolate was able to grow preferentially in feather meal broth (FMB) substrate and showed high proteolytic activity at temperatures of approximately 20°C, within the range of psychrophilic microorganisms (3). Considering its biotechnological potential, mainly due to its production of various extracellular proteases, the genome sequence of the Lysobacter sp. A03 strain was obtained and the preliminary analysis is presented here.

A03 whole-genome shotgun sequencing was performed on the MiSeq Illumina platform using the MiSeq reagent kit, version 2. A total of 109,889 paired-end reads were obtained, with an average length of 240 bp and approximately 18-fold coverage. The assembly was performed using CLC Genomics Workbench (http://www.clcbio.com/products/clc-genomics-workbench/), A5-miseq (4), CISA (5), and SPADES (6), and considering the lower *N*_50_ value (51,277) and the smaller number of contigs (101), the assembly constructed by SPADES was chosen. The CheckM (7) program was used to assess the quality of the microbial genome, and the automatic annotation of the genome sequence was performed in the RAST server (8).

The draft genome sequence of strain A03 comprised 2,873,548 bp representing approximately 99.1% of the genome size, with a G+C content of 65.79%. A total of 2,615 coding sequences (CDSs), 46 tRNA genes, and 2 rRNA genes were predicted. As expected, many peptidase-coding genes were found, including a sequence coding for an extracellular keratinase; furthermore, three genes involved in cold shock response (2 *cspA* and 1 *cspG*) and six beta-lactamase resistance genes were predicted.

The availability of the genome sequence from Lysobacter sp. A03 may provide methods for searching for new biotechnologically relevant enzymes and might increase the understanding of the possible mechanisms that lead to the cold adaptation of life.

### Nucleotide sequence accession numbers.

This whole-genome shotgun project has been deposited at GenBank under the accession no. JXSS00000000. The version described in this paper is version JXSS01000000.
